# The Combined Treatment with Chemotherapeutic Agents and the Dualsteric Muscarinic Agonist Iper-8-Naphthalimide Affects Drug Resistance in Glioblastoma Stem Cells

**DOI:** 10.3390/cells10081877

**Published:** 2021-07-24

**Authors:** Claudia Guerriero, Carlo Matera, Donatella Del Bufalo, Marco De Amici, Luciano Conti, Clelia Dallanoce, Ada Maria Tata

**Affiliations:** 1 Department of Biology and Biotechnologies Charles Darwin, Sapienza University of Rome, 00185 Rome, Italy; claudia.guerriero@uniroma1.it; 2 Department of Pharmaceutical Sciences, Medicinal Chemistry Section “Pietro Pratesi”, University of Milan, 20133 Milan, Italy; carlo.matera@unimi.it (C.M.); marco.deamici@unimi.it (M.D.A.); clelia.dallanoce@unimi.it (C.D.); 3 Preclinical Models and New Therapeutic Agents Unit, Regina Elena National Cancer Institute, 00187 Rome, Italy; donatella.delbufalo@ifo.gov.it; 4 Department of Cellular, Computational and Integrative Biology—CIBIO, University of Trento, 38123 Trento, Italy; luciano.conti@unitn.it; 5 Research Centre of Neurobiology Daniel Bovet, 00185 Rome, Italy

**Keywords:** M2 muscarinic receptor, glioblastoma, cancer stem cells, drug resistance, efflux pumps, chemotherapy

## Abstract

Background: Glioblastoma multiforme (GBM) is characterized by heterogeneous cell populations. Among these, the Glioblastoma Stem Cells (GSCs) fraction shares some similarities with Neural Stem Cells. GSCs exhibit enhanced resistance to conventional chemotherapy drugs. Our previous studies demonstrated that the activation of M2 muscarinic acetylcholine receptors (mAChRs) negatively modulates GSCs proliferation and survival. The aim of the present study was to analyze the ability of the M2 dualsteric agonist Iper-8-naphthalimide (N-8-Iper) to counteract GSCs drug resistance. Methods: Chemosensitivity to M2 dualsteric agonist N-8-Iper and chemotherapy drugs such as temozolomide, doxorubicin, or cisplatin was evaluated in vitro by MTT assay in two different GSC lines. Drug efflux pumps expression was evaluated by RT-PCR and qRT-PCR. Results: By using sub-toxic concentrations of N-8-Iper combined with the individual chemotherapeutic agents, we found that only low doses of the M2 agonist combined with doxorubicin or cisplatin or temozolomide were significantly able to counteract cell growth in both GSC lines. Moreover, we evaluated as the exposure to high and low doses of N-8-Iper downregulated the ATP-binding cassette (ABC) drug efflux pumps expression levels. Conclusions: Our results revealed the ability of the investigated M2 agonist to counteract drug resistance in two GSC lines, at least partially by downregulating the ABC drug efflux pumps expression. The combined effects of low doses of conventional chemotherapy and M2 agonists may thus represent a novel promising pharmacological approach to impair the GSC-drug resistance in the GBM therapy.

## 1. Introduction

Glioblastoma multiforme (GBM) is the most malignant form of human brain cancer. Considering the expression of some markers and GBM molecular properties, it has been hypothesized that GBM may originate either from neural stem cells (NSCs), or astrocytes or oligodendrocyte precursor cells (OPCs) [1,2,3]. Statistical reports from the Central Brain Tumor Registry of the United States have shown that GBM counts the highest number of cases of all malignant brain tumors, with about 12,900 cases in 2019 and 13,140 in 2020. The median survival rate is 14–15 months after initial diagnosis and fewer than 6% of patients survive for 5 years [4]. GBM is characterized by cellular and morphological heterogeneity, due to the coexistence of different cell populations with various grades of differentiation and distinct genetic and epigenetic profiles [5,6]. This tumor heterogeneity plays a crucial role in tumor growth, progression, invasiveness, and resistance to therapy [7]. In the last years, the theory that cancer stem cells are involved in the initiation and evolution of the tumors has emerged. These cells, through asymmetric divisions, on one side maintain their stemness characteristics by preserving their subpopulation and, on the other, generate more differentiated daughter cells that will constitute the majority of the tumor bulk [7]. According to this theory, GBM stem cells (GSCs) might represent the tumorigenic cell component in the brain tumors [8], albeit the tumor recurrence may also be dependent on the high invasiveness of these tumor cells and the great difficulty in a complete surgical excision.

Following orthotopic transplantation, GSCs are also able to generate tumor phenocopies that recapitulate the heterogeneity present in the parental tumor [8,9]. Notably, GSCs are grown in the same culture conditions of NSCs, with which they share similarities in markers expression and genetic profile, supporting the concept that GSCs may originate from transformed NSCs [10,11,12]. Currently, GBM treatment consists in surgery followed by adjuvant radiotherapy and chemotherapy based on the alkylating agent temozolomide [12,13]. In 90% of cases, a tumor recurrence is observed, since the current therapies are unable to accomplish a complete eradication of GSCs [14]. Nowadays, one of the greatest challenges in GBM treatment is to counteract the resistance to chemotherapy. Multidrug resistance (MDR) is a complex and multifactorial phenomenon involving a variety of mechanisms. In GBM, high MDR expression has been observed especially in GSCs. As a matter of fact, this cell subpopulation, resisting a wide variety of chemotherapeutic agents, survives and repopulates the tumor bulk [15,16]. One of the well-characterized causes of MDR implies the increased expression of members of the ATP-binding cassette (ABC) transporter superfamily [17,18], which are transmembrane proteins functionally involved in a variety of physiological roles. ABC transporters have been shown to be over-expressed in tumor cells, causing a greater efflux of various chemotherapeutic compounds from cells. Therefore, promoting ABC efflux pumps inhibition constitutes an attractive strategy for antitumoral therapeutic intervention [19].

Muscarinic acetylcholine receptors (mAChRs) are G protein-coupled receptors found to be expressed in several primary and metastatic tumors. They play a role in the regulation of cell proliferation and migration of tumor cells (e.g., breast cancer [20], ovarian cancer [21], lung cancer [22], astrocytoma [23]). We have previously shown that GBM express several mAChRs [24] and focused on the M2 subtype that is classically an inhibitory receptor [25]. We demonstrated that the selective stimulation of M2 mAChRs by the orthosteric agonist Arecaidine Propargyl Ester (APE) decreased cell proliferation and survival, causing an arrest of the cell cycle both in GBM cell lines and in GSCs [24,26,27,28].

In addition to the orthosteric binding site, mAChRs host allosteric binding sites located in the extracellular domains [29,30]. The different muscarinic subtypes show a high degree of sequence homology in the orthosteric binding site domain that hinders the selectivity of orthosteric ligands. With a view to designing compounds for therapeutic purposes, orthosteric muscarinic agonists are usually endowed with poor selectivity and affinity, with consequent putative side effects due to the high dose required for receptor activation [31]. During the last decade, a new approach in drug design was developed, since a series of compounds, called ‘dualsteric ligands’, were synthesized and pharmacologically investigated. These molecules are hybrid derivates, containing the substructures of both orthosteric and allosteric ligands bridged by a spacer chain [32,33,34,35,36]. Iper-8-naphthalimide, identified as N-8-Iper, is one of the most effective dualsteric agonists for the M2 receptor previously characterized by our research group [37,38]. N-8-Iper consists of a pharmacophoric portion of the allosteric inverse agonist Naphmethonium linked by a flexible octamethylene chain to the orthosteric superagonist Iperoxo [39,40,41,42]. In a previous study, we demonstrated that N-8-Iper reduced GSC proliferation in a comparable manner to APE in GB7 cells. Interestingly, N-8-Iper showed a significant ability to reduce cell proliferation also at lower doses than APE. At the same dose as APE, N-8-Iper not only reduced cell growth but also caused cell death [43]. Our more recent studies have shown that M2 receptor activation by APE caused a downregulation of the ATP-binding cassette (ABC) efflux pumps expression in neuroblastoma and in breast cancer [44,45]. Furthermore, co-treatment with chemotherapy drugs and M2 mAChR agonists significantly counteracts cell proliferation when compared with the single treatment [44]. Based on these results, in the present work we firstly investigated the ability of N-8-Iper to modulate cell proliferation in another GSC line (G166 cells) in order to extend the previous observation obtained in a single cell line (GB7). Then, we identified in both cell lines the dose of N-8-Iper that resulted as ineffective in terms of cell growth. The subtoxic doses for three selected chemotherapeutic agents (doxorubicin, cisplatin, and temozolomide) were also identified. The action of the M2 dualsteric agonist N-8-Iper in combination with each chemotherapeutic agent at a low dose was also tested, in order to determine whether combined treatment may exhibit a synergistic effect in reducing tumor cell growth. The results obtained suggest that activation of the M2 mAChR by N-8-Iper produces an increased sensitivity of GSCs to treatment with low-doses of conventional chemotherapy drugs. Moreover, our data suggest that the downregulation of the expression of ABC efflux pumps, which are typically over-expressed in GSCs, may be one of the mechanisms useful in counteracting chemoresistance.

## 2. Materials and Methods

### 2.1. Cell Cultures

The GSC lines GB7 and G166 were obtained from human GBM biopsies [9,46,47]. The cells were cultured on laminin-coated dishes (1 μg/mL; Sigma-Aldrich, St. Louis, MO, USA) or as neurospheres (on uncoated plastic) and maintained in serum free medium consisting of DMEM/F12 (1:1; v:v) (Corning, New York, NY, USA) supplemented with 1% streptomycin, 50 IU/mL penicillin (Sigma-Aldrich, St. Louis, MO, USA), 1% glutamine (Sigma-Aldrich, St. Louis, MO, USA), 1% N2 supplement (Invitrogen, Monza, Italy), 2% B27 (Invitrogen, Monza, Italy), 20 ng/mL EGF (Recombinant Human Epidermal growth factor, Peprotec, London, UK), and 20 ng/mL FGF (Recombinant Human FGF-basic, ABM, Richmond, Canada). The cultures were maintained at 37 °C in an atmosphere of 5% CO_2_/95% air. Human glioblastoma U251MG cell line was cultured in DMEM (Sigma-Aldrich, St. Louis, MO, USA) plus 10% fetal bovine serum (Sigma-Aldrich, St. Louis, MO, USA), 50 µg/mL streptomycin, 50 IU/mL penicillin, 2 mM glutamine (Sigma-Aldrich, St. Louis, MO, USA), 1% non-essential amino acids (Sigma-Aldrich, St. Louis, MO, USA), and maintained at 37 °C in a 10% CO_2_ atmosphere.

### 2.2. Pharmacological Treatment

Iper-8-naphthalimide (N-8-Iper) was synthesized according to published procedures [41] and the analytical features of the newly prepared compound were identical to those reported previously. N-8-Iper was used to activate M2 mAChR, since its ability to selectively bind this receptor subtype was demonstrated in GSCs by pharmacological binding experiments and knockdown of the M2 receptors by the siRNA transfection pool [43].

### 2.3. Cell Viability Assay

Cells were seeded on laminin-coated 96-well plates at the density of 2 × 104 cells/well for the GB7 cells and at the density of 1 × 104 cells/well for the G166 cells. After 24 h, cells were treated with N-8-Iper at different times (ranging from 72 to 120 h). Cell proliferation was evaluated by colorimetric assay based on 3-(4,5-dimethylthiazol-2-y1)-2,5-diphenyltetrazolium bromide (MTT, Sigma-Aldrich, St. Louis, MO, USA) metabolization. The MTT assay was performed according the protocol optimized by Mosmann [48]. MTT was dissolved in PBS at 5 mg/mL. The MTT stock solution (10×) was added and diluted (1×) in each well and then incubated at 37 °C for 3 h. Isopropanol (+ HCl 0.04 M + 1% Triton X-100) was added to all wells and mixed thoroughly to dissolve the dark blue crystals. For each well, the optical density (OD) at 570 nm was measured by Multiskan FC (Thermofisher Scientific, Waltham, MA, USA).

### 2.4. Chemosensitivity Test

To evaluate the chemosensitivity of both cell lines at different concentrations of the chemotherapy drugs, we performed chemosensitivity tests, treating cells with decreasing concentrations of temozolomide, cisplatin, and doxorubicin. In this regard, our experiments were based on the treatment of GB7 and G166 cells with decreasing concentrations of doxorubicin (range 100–6.25 nM), cisplatin (range 25–0.5 μM for GB7 cells; range 50–3.12 μM for G166 cells), and temozolomide (range 50–3.12 μM) for 72–96–120 h. The data obtained allowed to identify the lowest concentration that did not show effects on cell proliferation in both cell lines. Therefore, for the following experiments, we selected the concentration of 12.5 nM for doxorubicin, 3 μM for cisplatin, and 3 μM for temozolomide for the G166 cells, and the concentration of 6.25 nM for doxorubicin, 0.5 μM for cisplatin, and 3 μM for temozolomide for the GB7 cells.

### 2.5. Quantitative Estimation of Doxorubicin Cell Accumulation

Doxorubicin has an intrinsic fluorescence detectable in the visible spectral region and it can be efficiently excited with a blue light source [49]. For a quantitative estimation of doxorubicin accumulation, cells were seeded on laminin-coated 12-well plates at the density of 2 × 10^5^ cells/well. The next day, cells were treated with doxorubicin (100 μM). After 1 h, cells were observed under a fluorescence microscope (Axioskop2, Zeiss, Oberkochen, Germany), using a 40× objective through the Axion Vision program (Zeiss, Oberkochen, Germany).

The culture media were then supplied with verapamil (100 μM) or N-8-Iper (1.5 μM for GB7 cells and 12.5 μM for G166 cells) where required. After 3 h, the treated cells were observed again, and representative fields were photographed by fluorescence microscope (Axioskop2, Zeiss, Oberkochen, Germany). The culture media were collected from each well and the fluorescence intensity was measured. At the same time, to assess the amount of doxorubicin accumulated into the cells, they were collected, and fluorescence was measured in the cell lysates. The fluorescence intensity for both media and lysates were measured by the GloMax Multi Detection System (Promega, Madison, WI, USA) (λex, 470 nm; λem, 590 nm). The lysates and the protein content were obtained as indicated below in the Western blot section.

### 2.6. RNA Extraction, RT-PCR and q-PCR

Total RNA was extracted using Tri-Reagent (Sigma-Aldrich, St. Louis, MO, USA). RNA concentration and purity were detected using the NanoDrop Lite Spectrophotometer (Thermo, Dreieich, Germany). For each sample, 1 μg of total RNA was reverse transcribed using 5x-All-In-One-RT-Mastermix (abm, Viking Way, Richmond, BC, Canada) following the manufacturer’ instructions. The expression of stem cell markers and M2 mAChR was evaluated by semi-quantitative RT-PCR. Primers and GoTaq Green Master Mix (Promega, Madison, WI, USA) were added to 100 ng of cDNA. Below are reported the sequences of the primers used for the RT-PCR:

***18S*:** forward, 5′-CCAGTAAGTGCGGGTCATAAGC-3′
reverse, 5′-AACGATCCAATCGGTAGTAGCG-3′
***Nestin*:** forward, 5′-GCAGTGTGCGTTAGAGGTGC-3′
reverse, 5′-TCCAGAAAGCCAAGAGAAGC-3′
***Sox2*:** forward, 5′-ACACCAATCCCATCCACACT-3′
reverse, 5′-GCAAACTTCCTGCAAGCTC-3′
***M2 mAChR*:** forward, 5′-CTCCAGCCATTCTCTTCTGG-3′
reverse, 5′-GCAACAGGCTCCTTCTTGTC-3′

The expression of the efflux pumps was analyzed by real time-PCR (RT-qPCR). Twenty nanograms of each cDNA was used as a template in each well of the plate for RT-qPCR assay. RT-qPCR was performed with SYBR Green Mastermix (Promega, MI, Italy) and primers (final concentration 200 nM) added at the respective wells and analyzed by Thermofisher Quantstudio3 (Waltham, MA, USA). Quantification was expressed as 2−∆∆CT, where ∆∆CT = ∆CTsample − ∆CTcalibrator. The primers used for RT-qPCR are the following:

***ABC B1*:** forward, 5′-CGACAGGAGATAGGCTGGTT-3′
reverse, 5′-AGAACAGGACTGATGGCCAA-3′
***ABC C1*:** forward, 5′-TGCTCACTTTCTGGCTGGTA-3′
reverse, 5′-ACAGGACCAGACGAGCTGAA-3′
***ABC C4*:** forward, 5′-AGACCCCAACTCTACAAGGC-3′
reverse, 5′-ATTCTTCCATGCACGCTGAC-3′
***ABC G2*:** forward, 5′-GGAACTCAGTTTATCCGTGG-3′
reverse, 5′-CGAGGCTGATGAATGGAGAAG-3′

### 2.7. Western Blot Analysis

Cells were harvested in lysis buffer (Tris-EDTA 10 mM, 0.5% NP40, NaCl 150 mM), containing a protease inhibitor cocktail (Sigma-Aldrich, St. Louis, MO, USA) for 20 min in ice. After protein extraction, the quantification of the total amount of protein was determined by the Pierce BCA Protein Assay Kit (Thermo Fisher Scientific, Waltham, Massachusetts, USA) according to the manufacturer’s protocol. Then, the sample buffer supplemented with 5% β-mercaptoethanol was added to protein lysates and heated for 5 min at 95 °C. The protein extracts were run on SDS-polyacrylamide gel (SDS-PAGE) and transferred to Polyvinylidene Difluoride (PVDF) sheets (Merck Millipore, Darmstadt, Germany). Membranes were blocked for 40 min in 5% of non-fat milk powder (Sigma-Aldrich, St. Louis, MO, USA) in PBS containing 0.1% Tween-20 and then incubated with the primary antibody anti-ChRM2 (Novus Biologicals, Centennial, CO, USA) overnight at 4 °C. Beta-actin (Immunological Sciences, Rome, Italy) was used as protein reference for loading control. The blots were then washed three times with PBS + 0.1% Tween-20, then incubated with secondary antibodies conjugated to horseradish-peroxidase for 1 h. The reaction was revealed by ECL chemiluminescence reagent (Immunological Science, Rome, Italy). The bands were detected by exposition to Chemidoc (Molecular Imager ChemiDoc XRS + System with Image Lab Software, Biorad, CA, USA).

### 2.8. Statistical Analysis

Student’s *t* test and one-way ANOVA test followed by Bonferroni’s post-test or Dunnett’s post-test were used to evaluate statistical significance within the different samples. The results were considered statistically significant at *p* < 0.05 (*), *p* < 0.01 (**), and *p* < 0.001 (***).

## 3. Results

### 3.1. Stemness Property of G166 and GB7 Cell Lines

**Figure 1:**
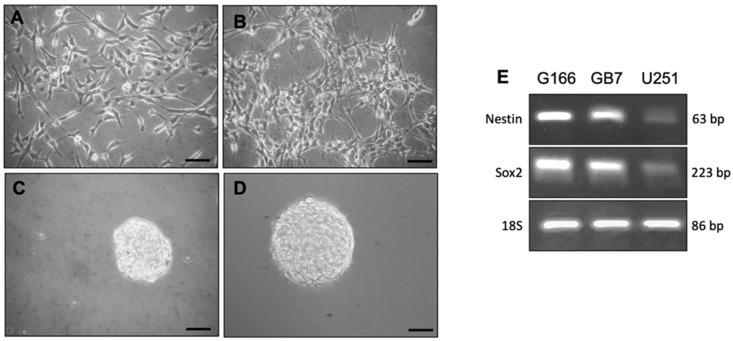
G166 (**A**) and GB7 (**B**) cells grown in monolayer conditions; G166 (**C**) and GB7 cells (**D**) grown as floating neurospheres. Scale bars: 50 μm. (**E**) RT-PCR analysis of stemness markers transcripts (*Nestin* and *Sox2*) expression in G166 and GB7 cells and in U251 line. 18S was used as housekeeping gene.

The two cell lines (G166 and GB7) were grown both in monolayers on laminin substrate (Figure 1A,B) and suspension as neurospheres (Figure 1C,D). The presence of Nestin and Sox2, two key neural stem cell markers, was evaluated by RT-PCR (Figure 1E). The GBM cell line U251 was used as a negative control. This analysis showed that the two GSC lines consistently express these markers; the U251 cells showed faint expression for these markers, due to their non-stem cell-like nature.

### 3.2. Expression of M2 mAChRs in GSCs and Analysis of Cell Growth

**Figure 2:**
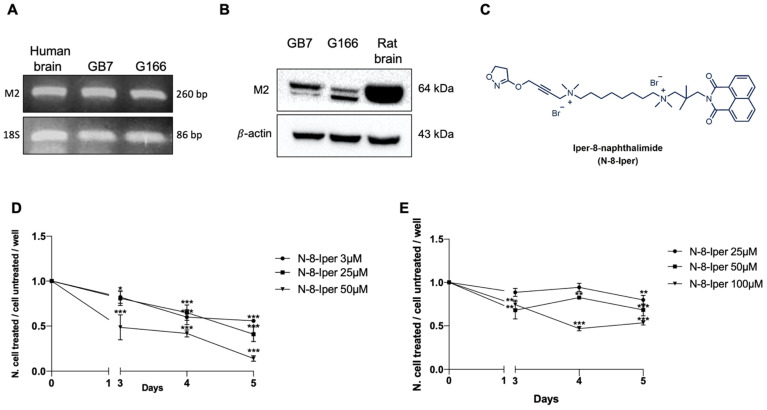
(**A**) RT-PCR analysis of *M2 mAChR* transcript expressed in GB7 and G166 cells. Human brain was used as a positive control. 18S was used as housekeeping gene; (**B**) M2 protein expression by Western blot analysis in GB7 and G166 cells. Rat brain was used as a positive control and β-actin was used as internal reference protein; (**C**) Chemical structure of the M2 dualsteric ligand, N-8-naphthalimide (N-8-Iper); (**D**) Effect of N-8-Iper (3, 25, 50 μM) on GB7 cell growth at different times of treatment (ranging from 72 to 120 h); (**E**) Effect of N-8-Iper (25, 50, 100 μM) on G166 cell growth at different times of treatment (ranging from 72 to 120 h). Data represent the average ± SEM of three different experiments performed in triplicate. ANOVA test was used followed by Dunnett’s test (N-8-Iper treated cells vs. respective control, *** *p* < 0.001; ** *p* < 0.01; * *p* < 0.05).

Expression of the M2 mAChR was investigated in G166 and GB7 cells. As shown in Figure 2A, RT-PCR analysis revealed the presence of *M2 mAChR* mRNA in both GSC lines. Western blot analysis confirmed that the M2 receptor protein was present in both GB7 and G166 cells (Figure 2B). However, in the two cell lines, the bands appeared at two different molecular weights. The GB7 cells, at variance with the G166 cells, displayed increased expression levels of the higher molecular weight band. On the other hand, G166 cells exhibited greater expression levels of the lowest molecular weight band. This result could be indicative of the presence of different levels of post-translational modifications (e.g., glycosylation) of the M2 mAChR in the two cell lines.

As previously determined for the GB7 cells [43], the selective activation of M2 receptors by the dualsteric agonist N-8-Iper (Figure 2C) caused a reduction in cell proliferation in a time- and dose-dependent manner. After 72 h treatment, the lowest concentration of N-8-Iper capable of producing a significant decrease of cell number was 3 μM [43]. By the MTT assay, we evaluated on the two cell lines the action of different concentrations of N-8-Iper on the two cell lines, following a 72, 96 and 120 h treatment. In view of previous results obtained for GB7 cells, we analyzed the anti-proliferative effects produced by the lowest dose of N-8-Iper (3 μM) as well as by a medium (25 μM) and a high (50 μM) dose (Figure 2D). In early analysis, G166 cells were found to be more resistant to N-8-Iper treatment than the GB7 cells. N-8-Iper produced significant anti-proliferative effects at selected concentrations, ranging from 25 μM to 100 μM (Figure 2E). At lower doses, no effect on cell growth reduction was appreciated on G166 cells (data not shown).

### 3.3. Chemosensitivity Assay

**Figure 3:**
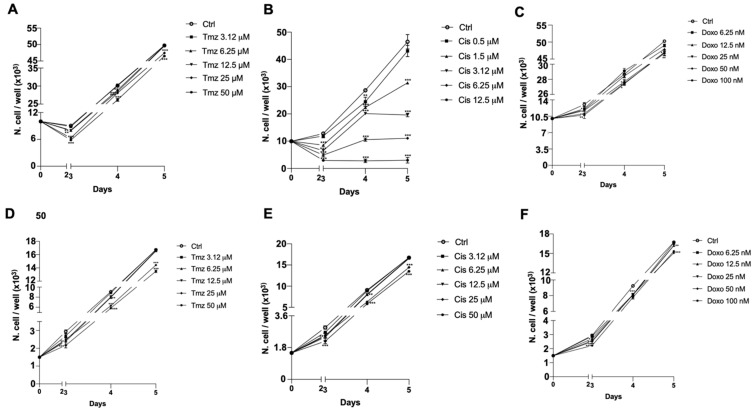
Analysis of cell survival by the MTT assay with temozolomide (Tmz) at concentrations ranging from 3.12 to 50 μM in GB7 (**A**) and G166 (**D**) cells, Cisplatin (Cis) at concentrations ranging from 0.5 to 25 μM in GB7 cells (**B**) and at concentrations ranging from 3.12 to 50 μM in G166 cells (**E**), and Doxorubicin (Doxo) at concentrations ranging from 6.25 to 100 nM in GB7 (**C**) and G166 (**F**) cells. Data presented are the average ± SEM of three independent experiments conducted in triplicate. ANOVA test was used followed by Dunnett’s test (Chemotherapy drugs treated cells vs. respective control, *** *p* < 0.001; ** *p* < 0.01; * *p* < 0.05).

To evaluate the pharmacological response of both GB7 and G166 cells to conventional chemotherapy drugs, such as temozolomide (Tmz), cisplatin (Cis) and doxorubicin (Doxo), we performed a chemosensitivity assay. This assay allowed us to identify the subtoxic doses of the three drugs unable to produce a significant reduction in cell number. The MTT assays were performed on both cell lines after 72, 96, and 120 h of treatment with decreasing concentrations of Tmz, Cis, and Doxo (Figure 3). G166 cells showed greater resistance to treatment with conventional chemotherapy drugs than the GB7 cells, this trend being more evident in Cis treatment (Figure 3B,E). We found that after 72 h of treatment, the doses of 3 μM Tmz (Figure 3A), 0.5 μM Cis (Figure 3B), and 6.25 nM Doxo (Figure 3C) did not show any effects on the reduction of cell growth in GB7 cells. In contrast, for the G166 cells, the subtoxic doses in terms of cell number reduction after 72 h of treatment were as follows: 3.12 μM Tmz (Figure 3D), 3.12 μM Cis (Figure 3E), and 12.5 nM Doxo (Figure 3F).

**Figure 4:**
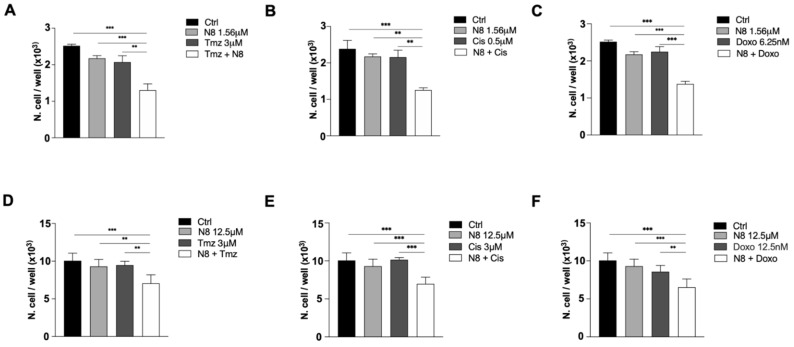
Analysis of cell survival by MTT assay after 72 h of co-treatment with low doses of N-8-Iper (1.56 μM) and Tmz 3 μM in GB7 (**A**) and G166 cells treated with N-8-Iper (12.5 μM) and Tmz 3 μM (**D**); Cis 0.5 μM in GB7 cells (**B**) and Cis 3 μM in G166 cells (**E**); and Doxo 6.25 nM in GB7 cells (**C**) and Doxo 12.5 nM in G166 cells (**F**). Data represented are the average ± SEM of three independent experiments conducted in triplicate. An ANOVA test was used followed by Bonferroni’s test to statistically compare all experimental conditions (*** *p* < 0.001; ** *p* < 0.01).

From the previous MTT assay (Figure 2D,E), we selected the first non-effective concentrations of the M2 agonist N-8-Iper: 1.56 μM for GB7 cells and 12.5 μM for G166 cells. As a first step, we assessed whether the non-effective dose of N-8-Iper, coupled with the subtoxic concentration of the chosen chemotherapeutic drugs, could exert a combined effect on cell survival. As shown in Figure 4, the single low-dose treatments did not produce any appreciable effect in terms of cell survival with respect to the control condition. In contrast, when we combined a low dose of N-8-Iper with one of the chemotherapeutic agents at low doses, we observed a significant decrease in cell growth compared to the untreated cells or to cells treated with Tmz, Cis, or Doxo alone.

### 3.4. Evaluation of Doxorubicin Cell Accumulation

**Figure 5:**
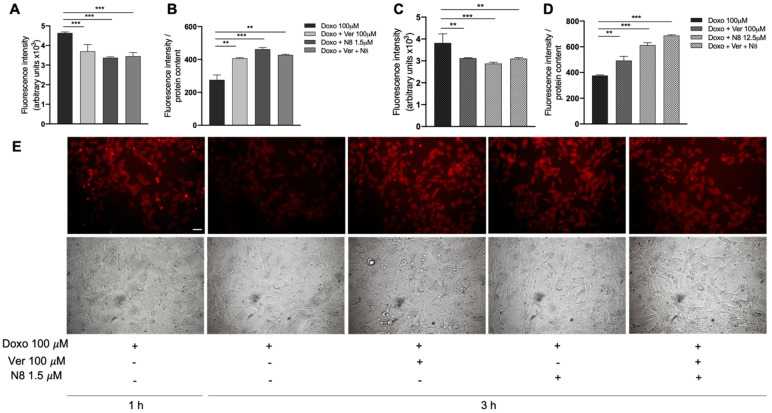
Analysis of the fluorescence intensity in culture media in GB7 (**A**) and in G166 (**C**) cells, and in cell lysates in GB7 (**B**) and in G166 (**D**) cells after 4 h of treatment with Doxo 100 µM. In the samples treated with verapamil (Ver 100 µM) or N-8-Iper (1.5 µM for GB7 and 12.5 µM for G166), the inhibitors were added after 1 h of Doxo treatment and maintained for an additional 3 h. Then the media and cells were collected and the fluorescence measured in media and in cell lysates. Data presented are the average ± SEM of three independent experiments conducted in triplicate. An ANOVA test was used followed by Bonferroni’s test to statistically compare all experimental conditions (*** *p* < 0.001; ** *p* < 0.01). (**E**) Representative pictures of the fluorescence derived from Doxo present into GB7 cells. The cells were maintained in the same experimental conditions described above and were those used in one of the experiments whose data are reported in panels (**A**) and (**B**), scale bar: 50 μm.

To assay the amount of drug internalized and held inside the cells in control conditions (Doxo 100 µM) and after treatments (Doxo + Verapamil; Doxo + N-8-Iper; Doxo + Verapamil + N-8-Iper), we exploited the intrinsic fluorescent properties of doxorubicin. After 1 h of treatment with Doxo 100 µM, 100 µM Verapamil (Ver), an inhibitor of drug efflux pumps [50], or N-8-Iper at low doses were added to the two cell lines. Three hours later, the culture media were removed, and the fluorescence was measured. Interestingly, the medium derived from the cells that were maintained in the presence of Doxo alone showed a higher fluorescence intensity than those from the cells maintained in the other experimental conditions (Doxo + Ver; Doxo + N-8-Iper; Doxo + N-8-Iper + Ver) (Figure 5A,C). The fluorescence intensity was also evaluated in the cell lysates. As shown in Figure 5B,D, in both cell lines, the cell lysates, obtained from cells maintained in the presence of Doxo alone, showed a lower fluorescence intensity than those kept in the other experimental conditions. These data clearly indicate that a higher amount of Doxo remains within the GSC cells when treated with Ver or N-8-Iper.

The above reported results were also confirmed by fluorescence microscopy analysis. As shown in Figure 5E, an accumulation of doxorubicin was observed in the GB7 cells after 1 h of Doxo treatment. The fluorescence decreased after an additional 3 h of Doxo treatment, indicating that an amount of drug was expelled from the cells. Conversely, in those cells treated with Ver or N-8-Iper (or both), the fluorescence intensity was comparable to that observed in the cells treated for 1 h with Doxo alone (Figure 5A). A similar trend was observed in the parallel experimental protocol applied to G166 cells (data not shown).

### 3.5. N-8-Iper Modulates the ABC Efflux Pumps Expression

**Figure 6:**
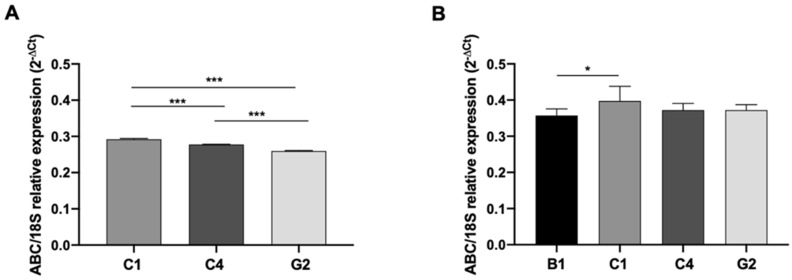
RT-qPCR analysis for *ABC-B1, ABC-C1, ABC-C4,* and *ABC-G2* transcript levels in GB7 (**A**) and G166 (**B**) cells. The data are the average ± SEM of three independent experiments performed in triplicate; Student’s *t*-test was used to statistically compare the different *ABC* pumps transcripts levels (*** *p* < 0.001; * *p* < 0.05).

As far as the ability of low doses of the studied M2 agonist to amplify the inhibitory effects of chemotherapeutic agents on GCS proliferation is taken into account, as well as its action in counteracting Doxo efflux, we investigated the modulation of the ABC efflux pumps by N-8-Iper in both GSC lines. At first, we characterized by RT-qPCR analysis the expression of *ABC-B1, ABC-C1, ABC-C4*, and *ABC-G2* pumps in the two GSC lines (Figure 6A,B). Evidence showed that G166 cells expressed all the considered efflux pumps, whereas the GB7 cells did not express the *ABC-B1* pump transcript.

**Figure 7:**
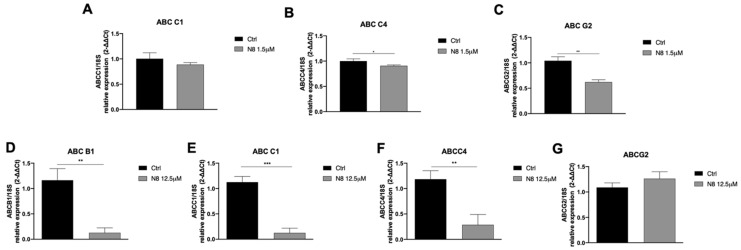
RT-qPCR analysis showing the *ABC-C1, ABC-C4,* and *ABC-G2* ((**A**), (**B**), (**C**), respectively) transcript levels in GB7 cells after 24 h treatment with 1.5 μM N-8-Iper, and the *ABC-B1, ABC-C1, ABC-C4,* and *ABC-G2* transcript levels ((**D**), (**E**), (**F**), (**G**), respectively) in G166 cells after 24 h treatment with 12.5 μM N-8-Iper. The data are the average ± SEM of three independent experiments performed in triplicate; Student’s *t*-test was used to statistically compare the different experimental conditions (*** *p* < 0.001; ** *p* < 0.01; * *p* < 0.05).

To evaluate a possible modulation of the efflux pumps following the M2 mAChR activation, we analyzed the transcriptional levels of the different ABC pumps after 24 h of treatment with the low-dose of N-8-Iper agonist (12.5 μM for G166 cells; 1.56 μM for GB7 cells). The treatment with 12.5 μM N-8-Iper was able to significantly downregulate the transcriptional expression of *ABC-B1, ABC-C1*, and *ABC-C4* pumps in G166 cells (Figure 7D–F), while the *ABC-C4* and *ABC-G2* pumps were downregulated in GB7 cells after the treatment with 1.56 μM N-8-Iper (Figure 7B,C).

## 4. Discussion

The contribution of muscarinic receptors to tumor progression was largely demonstrated. In this research field, our group has been investigating in depth the role of the M2 mAChRs in various tumors (e.g., neuroblastoma, breast cancer, and urothelial cancer cells), including GBM [24,26,27,28,51]. Collectively, these studies confirm the relevance of this muscarinic receptor subtype to counteract the cell growth in different tumor types. Indeed, selective stimulation of the M2 receptor by APE in GSCs affected cell proliferation and survival, albeit at high doses (50–100 μM). Despite this promising profile of the orthosteric agonist, APE indicated the M2 mAChR as a potential target for GBM; however, more powerful, safe, and selective agonists are desirable in view of therapeutic applications. To this aim, in recent years, we analyzed the effects of a new muscarinic ligand, N-8-Iper, which is a potent bitopic agonist able to selectively activate the M2 receptors [43]. Our results showed that N-8-Iper acts as a more potent agonist than APE, most likely due to its ability to bind not only the orthosteric site, like APE, but also the allosteric recognition site of the M2 receptor. Moreover, N-8-Iper efficiently inhibited cell growth at lower doses than APE [43]. Based on these promising data, in this work we aimed at better investigating the effects produced by this bitopic muscarinic agonist on the control of cell proliferation and chemoresistance in GBM. Since our recent results give evidence that APE was able to counteract drug resistance in neuroblastoma and in breast cancer cell lines [44,45], we planned to perform similar experiments in two GBM GSC lines (GB7 and G166 cells), obtained from two different human GBM biopsies [52]. As described by Baronchelli and colleagues. [52], these GSC lines have different cytogenomic and epigenomic profiles. Among the various alterations, G166 cells showed the gain of EGFR and MDM4 genes and the whole chromosome X. On the other hand, GB7 cells were characterized by the complete loss of chromosome 10, leading to a lack of PTEN and DMBT1, and by the loss of 9p21.3 locus, containing CDKN2A and CDKN2B genes. The neural stem cell properties of the two GSC lines were initially confirmed by evaluating the expression of stem cell markers and the ability of these cells to grow in monolayers or in suspension conditions (see Figure 1).

M2 mAChR expression in the two different GSC lines was assayed by RT-PCR and Western blot analysis. Evaluation of the transcript levels showed that both cell lines expressed M2 receptor mRNA and no significant differences in the expression level were observed (Figure 2A). Interestingly, analysis of the M2 receptor at the protein level showed differences between the two cell lines. In GB7 cells, the higher weight band was more expressed, whereas in G166 cells, the lower weight band was found to be more intense (Figure 2B). Albeit these data deserve further investigation, Western blot analysis could indicate a different level of modifications (e.g., the extent of glycosylation) of the receptor in the two cell lines, suggesting that the GB7 cells may express more glycosylated M2 receptors than G166 cells. This might partly explain why the two GSC lines respond differently to N-8-Iper, in terms of cell growth, indicating a higher resistance to the treatment with M2 agonist in G166 cells. Previous data on GB7 cells showed that N-8-Iper reduced cell growth in a dose-dependent manner, highlighting its ability to act at much lower concentrations (3 μM) than those of the orthosteric agonist APE [43]. Based on these results, the anti-proliferative effects of M2 activation via N-8-Iper in G166 cells were analyzed and compared with the already known effects on GB7 cells (Figure 2D,E). The MTT assay showed increased resistance to treatment with N-8-Iper of the G166 cells, since the first dose of N-8-Iper able to impair cell growth was 25 μM. This variation in response to muscarinic stimulation may be in part caused by a different genetic background of the cells but may be also due to the different expression levels and glycosylation state of M2 receptor and/or to a difference in affinity or efficacy of N-8-Iper for the M2 receptors expressed in these cells.

In the last two decades, a great deal of progress has been made in understanding the mechanisms by which cancer cells become resistant to chemotherapy. Genomic aberrations, elevated levels of enzymes involved in intracellular drug metabolism, deregulation of membrane transport proteins, and evasion of the apoptotic process are among the cellular mechanisms that cause chemotherapy treatment failure [53]. In recent years, a new chemotherapy regimen has emerged, called Metronomic Chemotherapy. It consists of frequent and regular administration of low doses of chemotherapeutic agents over longer periods of time, thereby significantly reducing side effects [54]. Recently, we demonstrated that the low doses of the muscarinic agonist APE in combination with low doses of the conventional chemotherapy drugs were able to produce a decrease of drug resistance in breast cancer [45] as well as in neuroblastoma [44] cells. Based on this evidence, here we tested the ability of low doses of the dualsteric M2 agonist N-8-Iper to affect GSC growth when combined with subtoxic doses of conventional chemotherapy drugs, such as temozolomide, cisplatin, and doxorubicin. To this end, we initially evaluated the chemosensitivity of two GSC lines, analyzing the cell growth by the MTT assay at different concentrations of temozolomide, cisplatin, and doxorubicin after 72, 96, and 120 h of treatment (Figure 3). The data obtained evidenced that GB7 cells respond to chemotherapy drugs in a dose-dependent manner, especially upon treatment with cisplatin and temozolomide. In contrast, G166 cells showed increased resistance to all three chemotherapeutic agents. However, through the chemosensitivity assay, we identified the subtoxic doses for all drugs used in combination with low doses of N-8-Iper in the two cell lines. The MTT assay clearly demonstrated that treatment with a low dose of N-8-Iper in combination with low doses of the chemotherapy drugs significantly decreased cell survival; conversely, when the drugs were used alone at the same doses, they did not produce any effects on GSCs growth compared to the control condition (Figure 4). The acquisition of a MDR phenotype is closely associated with the overexpression of drug transporters, such as the ATP-binding cassette (ABC) transporter superfamily. High expression of MDR-related genes, including ABC efflux pumps, is a well-known feature of cancer stem cells [55]. Based on this evidence, we moved to evaluate whether the M2 activation via the selective agonist N-8-Iper could mimic the effect of verapamil, an FDA-approved pump inhibitor [56]. Our experiments relied on the intrinsic fluorescent properties of doxorubicin, which allowed to assess whether the muscarinic agonist could affect the efflux of the drug from the cells, such as Verapamil, a blocker of the efflux pumps that causes drug retention within the cells [50,57]. After 1 h of treatment with doxorubicin, we added verapamil and a low-dose N-8-Iper. After 3 h, fluorescence intensity analysis of the media in the different experimental conditions revealed an increased amount of extruded doxorubicin in media derived from cells treated with doxorubicin alone with respect to the one from cultures treated with Verapamil and/or N-8-Iper (Figure 5A,C). According to these results, the fluorescence analysis of the corresponding cell lysates showed a lower amount of the fluorescence in cells treated only with doxorubicin compared to cells under the other experimental conditions (Figure 5B,D). These results were confirmed by the fluorescence images of the cells maintained in the same experimental conditions. Overall, our data corroborate the hypothesis that N-8-Iper impairs the efflux of the chemotherapeutic agents from the cells, thus exerting an effect similar to that of verapamil.

The analysis of the expression of *ABC* efflux pumps by qRT-PCR, upon low doses of chemotherapy drugs alone or in combination with low doses N-8-Iper, highlighted that M2 receptor activation significantly reduced the expression of several *ABC* efflux pumps (i.e., *ABC-C4* and *ABC-G2* in GB7 cells; and *ABC-B1*, *ABC-C1*, and *ABC-C4* in G166 cells). However, qRT-PCR revealed that the most resistant G166 cells showed a more pronounced downregulation of the efflux pumps than GB7 cells after low-dose N-8-Iper treatments. However, in addition to the downregulation of the activity and/or the expression of *ABC* efflux pumps, we cannot exclude that the M2 dualsteric agonist may modulate additional MDR processes, such as alteration of DNA mechanism repairs (i.e., the base excision repair (BER) pathway or the DNA mismatch repair (MMR) pathway) [58,59].

## 5. Conclusions

The results of this study confirm that the M2 agonist N-8-Iper counteracts cell growth in different GSCs. This bitopic ligand was found to be more potent than the reference orthosteric agonist APE, which makes it an attractive compound in view of potential therapeutic applications. Indeed, these outcomes, along with those from other studies on various tumor types [43,53], represent a good starting point to further deepen the role of M2 mAChRs in the cancer research field. Interestingly, additional data on glioblastoma, neuroblastoma, and breast cancer showed that M2 receptor activation counteracts drug resistance. In particular, a low dose of N-8-Iper may enhance the action of chemotherapy drugs at doses that alone would be ineffective. These data suggest that the metronomic chemotherapy coupled with a drug combination regimen may represent a new promising therapeutic approach for the cancer treatment, probably reducing the side effects produced by conventional chemotherapy.

## Data Availability

Not applicable.
